# Tailored exercise interventions to reduce fatigue in cancer survivors: study protocol of a randomized controlled trial

**DOI:** 10.1186/s12885-018-4668-z

**Published:** 2018-07-24

**Authors:** Rosie Twomey, Tristan Martin, John Temesi, S. Nicole Culos-Reed, Guillaume Y. Millet

**Affiliations:** 1 Faculty of Kinesiology, University of Calgary, 2500 University Dr NW, Calgary, AB T2N 1N4 Canada; 2 Department of Oncology, Cumming School of Medicine, University of Calgary, 2500 University Dr NW, Calgary, AB T2N 1N4 Canada

**Keywords:** Central fatigue, Peripheral fatigue, Sleep, Transcranial magnetic stimulation

## Abstract

**Background:**

Cancer-related fatigue (CRF) is a common and distressing symptom of cancer and/or cancer treatment that persists for years after treatment completion in approximately one third of cancer survivors. Exercise is beneficial for the management of CRF, and general exercise guidelines for cancer survivors are available. There are multiple potential pathways by which exercise improves CRF, and cancer survivors with CRF are diverse with respect to cancer type, treatments and experienced side effects. While the general exercise guidelines are likely sufficient for most cancer survivors, tailoring of exercise interventions may be more effective in those with persistent fatigue. The primary aim of this research is to investigate the effect of a traditional vs. tailored exercise intervention on CRF severity in cancer survivors with persistent CRF.

**Methods/design:**

Cancer survivors (≥ 3 months and ≤ 5 years since primary treatment) who score ≤ 34 on the Functional Assessment of Chronic Illness Therapy Fatigue Scale (FACIT-F) will be randomly allocated to one of two parallel treatment arms: traditional (active control) and tailored exercise. Participants in the traditional exercise group will engage in aerobic and resistance exercise that is consistent with exercise guidelines for cancer survivors. The tailored exercise group will be prescribed an intervention designed to address individual deficits identified at baseline, such as loss of muscular strength, cardiorespiratory deconditioning or sleep disturbance. Participants will be assessed before and after the intervention for CRF severity and other patient-reported outcomes, neuromuscular function and fatigue in response to whole-body exercise, sleep quantity and quality, physical activity levels, cardiorespiratory fitness and blood biomarkers.

**Discussion:**

To our knowledge, this will be the first study to compare the effects of a traditional vs. tailored exercise intervention on CRF severity in cancer survivors with persistent CRF. Using physiological, behavioural and patient-reported outcomes, this study will add to the current knowledge about both the factors contributing to CRF, and the potential reduction in CRF severity with an exercise intervention.

**Trial registration:**

The study is registered at ClinicalTrials.gov (NCT03049384), February, 2017.

**Electronic supplementary material:**

The online version of this article (10.1186/s12885-018-4668-z) contains supplementary material, which is available to authorized users.

## Background

Fatigue, characterised by a subjective sense of tiredness, is a common and distressing symptom associated with cancer or cancer treatment [1]. Cancer-related fatigue (CRF) differs from fatigue experienced by healthy individuals (e.g. following intense or prolonged exercise, or sleep deprivation), in that it is non-transient and less likely to be relieved by rest. With chronic fatigue, which is a hallmark of multiple pathologies, daily activities can be limited or associated with undue effort [2]. This is the case for CRF, which has been defined from an experiential perspective as a distressing, persistent sense of physical, emotional, and/or cognitive tiredness or exhaustion that is not proportional to recent activity and interferes with usual functioning [3]. Because there is no objective surrogate measure of CRF in humans, its severity is assessed as a patient-reported outcome. A number of CRF scales have been used for this purpose, and the Functional Assessment of Chronic Illness Therapy Fatigue Scale (FACIT-F) [4], a 13-item self-report questionnaire which delineates the physical and functional consequences of CRF, is widely recommended [5]. Alongside self-report, measures of physiological, behavioural and/or psychosocial ‘performance’ can supplement our understanding of fatigue as a symptom [6].

The majority of cancer patients will experience CRF during primary treatment, and this often improves or returns to baseline levels after treatment completion. However, one-third of cancer survivors are estimated to have clinically significant CRF which persists for months and years after cancer treatment [7]. This estimate of CRF is predominantly from breast cancer survivors [8], and it may be higher in other tumor groups (e.g. [9]). Considering the incidence of cancer (for example, 206,200 new cases were estimated for 2017 in Canada) and 60% five-year survival estimates (from people diagnosed between 2006 and 2008) [10], the prevalence of cancer survivors with CRF is likely to increase. CRF results in increased utilization of health care resources [11], impacts return to work, and reduces the capability to work [12]. Furthermore, CRF leads to a reduction in the health-related quality of life (HRQL) of cancer survivors [13]. Accordingly, refining or developing evidence-based interventions to alleviate CRF and its impact on functioning and HRQL is a priority for future research [14, 15].

Although the etiology of CRF is under investigation, recent evidence indicates a number of possible mechanisms. In the past decade, the pathogenic processes associated with CRF during or after treatment have been investigated using blood biomarkers and genomic variates [16, 17]. This research has led to the hypothesis that CRF involves multiple (and interacting) biological processes that result from cancer and/or cancer treatment [16]. These include alterations in the cellular immune response, hypothalamic–pituitary–adrenal axis dysfunction and inflammation [18, 19]. A number of physiological alterations and a resultant decline in physical function may be related to CRF, where cancer cachexia [20], other neuromuscular complications [21] and cardiorespiratory deconditioning [22] may contribute. These may be induced by cancer and/or cancer treatment and compounded by physical inactivity during/after treatment (where physical inactivity may also be a behavioural mediator of other alterations such as weight gain). CRF is also influenced by psychosocial factors, such as self-efficacy [23]. Furthermore, cancer survivors with CRF may also be experiencing chronic sleep disturbance as part of a multi-symptom cluster [24]. Given this range of potential contributing factors, investigating, and indeed treating the cause of CRF is challenging. There is minimal evidence of efficacy for any pharmacological treatment for CRF after cancer treatment [25]. Clinicians are guided to screen for a primary cause in the event that CRF is secondary to, for example, anemia, mood disorders or pain [26, 27]. However, where CRF is not resolved in this way (i.e. with treatment of a co-morbidity) and does not recover in the first few months after treatment, it may persist as a primary symptom indefinitely.

A number of behavioural interventions have been investigated for the improvement of CRF in adults (e.g. [28]), but there is widespread agreement the most beneficial intervention is exercise ([29–31]). However, a systematic review of several meta-analyses recently highlighted that although effects are in the direction of benefit, the magnitude of the effect of exercise on CRF varies substantially, such that caution is warranted when drawing definitive conclusions about exercise and CRF [32]. CRF is often a secondary rather than primary outcome of randomized controlled trials, and participant level of CRF at baseline is not necessarily clinically relevant, meaning that the effect of exercise on CRF may actually be diluted [32]. In addition, exercise interventions may not be optimally designed with an improvement in CRF as the target. Nevertheless, due to numerous health benefits, exercise should be part of standard care for cancer survivors, and the American College of Sports Medicine (ACSM) has published specific recommendations for exercise and CRF [33]. In accordance with other published guidelines [34–37], a combination of aerobic and resistance training is recommended, and the health-related physical activity guidelines for the general population are considered appropriate for most cancer survivors (with modifications as necessary for cancer-related side effects). However, it has also been suggested that this ‘one size fits all’ approach may be too generic, particularly in regards to the treatment of persisting side effects such as CRF [38].

Given the lack of pharmacological targets for CRF, and the wider evidence base for exercise as medicine in chronic diseases [39], the potential for improvement in CRF with exercise warrants further evaluation. CRF has been reported in many tumour groups, following a range of (often multi-modal) cancer treatments, meaning that it is likely that cancer survivors present with diverse physiological profiles (and/or deficits) prior to beginning an exercise intervention. Therefore, improvement in specific physiological parameters on an individual basis (such as cardiorespiratory fitness [40]) is a potential target for optimizing the improvement in CRF with exercise. By prescribing an intervention that is tailored to the individual and based on a range of pre-intervention data, the effectiveness of the intervention for the improvement in CRF may be enhanced. The initial data could also include an assessment of baseline sleep quality and quantity, where the potential reciprocal relationship between CRF and sleep disturbance deserves attention because an improvement in CRF with exercise may be related to improvements in sleep [23]. To date, the effect of exercise on sleep in cancer survivors has primarily been assessed using self-report questionnaires, despite differences when compared to objective measures such as actigraphy [41]. Few studies have identified cancer survivors with both CRF and sleep disturbance at baseline prior to an exercise intervention [42]. As such, the relationship between sleep disturbance, CRF and exercise is yet to be fully elucidated [43].

Although some physiological parameters have been associated with CRF, the neuromuscular correlates of CRF have received less attention. Fatigue has been considered under two distinct domains: the perceptions of fatigue (subjective sensations as in CRF) and performance fatigability (objective changes in task performance) [44, 45]. These domains are not mutually exclusive, and investigating performance fatigability may inform the discussion about the improvement of CRF with exercise [46]. One method used to investigate performance fatigability involves firstly measuring the force-generating capacity of a muscle or muscle group, as part of an assessment of neuromuscular function. Next, the participant performs a motor task that results in a reduction in force-generating capacity, i.e. neuromuscular fatigue. In combination with electrical and magnetic stimulation techniques, the central and peripheral contributions to neuromuscular fatigue can be appraised [47]. One such technique is transcranial magnetic stimulation (TMS), a non-invasive and safe method of brain stimulation that is widely used to stimulate the motor cortex in clinical settings [48]. TMS has been used to assess central alterations and fatigue in other chronic conditions [49–51], but to our knowledge, has not yet been used as an investigative tool in cancer survivors.

The measurement of neuromuscular function or fatigue in cancer survivors is relatively rare, with < 10 studies published on the topic [52–59]. The majority of these investigated participants with advanced cancer, who were on [56–58] or off [52–54] active treatment. To summarise the findings, these studies provide evidence that (i) cancer survivors with CRF are unable to sustain submaximal sustained [52–54] or intermittent [55, 56] isometric contractions for as long as matched controls; (ii) early task disengagement occurs with less evidence of peripheral fatigue (i.e. less disturbance to muscle contractile properties) [52, 54, 55] and (iii) central mechanisms may contribute more to the decision to terminate exercise in cancer survivors with CRF compared to matched controls [52, 53]. The evidence for the greater contribution of central mechanisms in these studies is mostly indirectly inferred [e.g. from electromyography or finding (ii)], and only one study [59] used the interpolated twitch technique to measure a reduction in voluntary activation [60] that is the current standard for the measurement of central fatigue. The majority of these studies used measurements in the upper limb [52–55, 58] and/or sustained isometric contractions as the motor task [52–54, 57, 59]. This is of limited functional relevance in regards to daily activities, particularly locomotion. In order to investigate dynamic exercise involving large muscle groups, our lab has developed an ergometer that allows a comprehensive measurement of neuromuscular fatigue before, during and immediately after (cycling) [61], which could be used to inform current understanding of the link between the neuromuscular system and CRF.

In summary, while generic exercise recommendations are likely sufficient for the majority of cancer survivors after treatment, tailoring of interventions may be warranted in cancer survivors with persistent CRF. Indeed, it has previously been suggested that interventions should be tailored according to the specific health outcome [62]. Here, we propose a comprehensive pre-intervention assessment including a number of pathways by which exercise may alleviate CRF, via improvements in physiological parameters or sleep. Based on objective assessments including cardiorespiratory fitness, neuromuscular function and sleep (actigraphy), a tailored exercise intervention will be designed based on individual deficits or areas for improvement. The primary aim of this research is to investigate the effect of a traditional vs. tailored 12-week exercise intervention on self-reported CRF severity (FACIT-F score) in cancer survivors with persistent CRF. We hypothesize that there will be an improvement in CRF after the exercise intervention in both groups, and that the improvement will be greater in the tailored vs. traditional exercise intervention.

## Methods/design

**Fig. 1:**
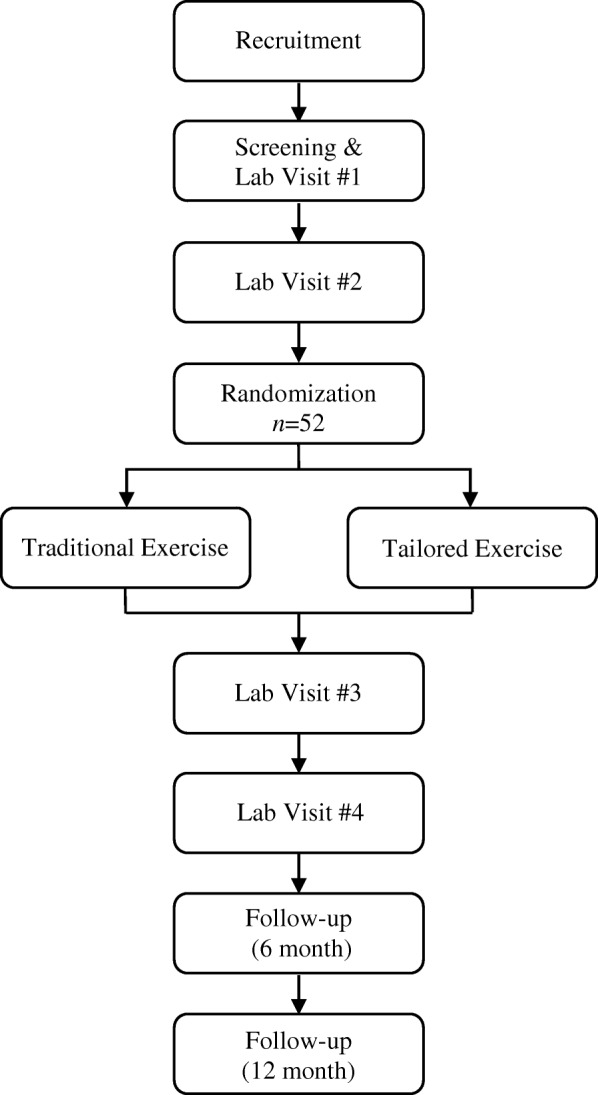
Flowchart of the study design

This study is a prospective randomized controlled trial with a two-armed parallel design and 1:1 allocation ratio. Flow through the study is presented in Fig. 1. This study has been approved by the Health Research Ethics Board of Alberta Cancer Committee (HREBA.CC-16-10-10). The approved study will be reviewed annually by the HREBA.CC until completion. Any amendment to the protocol which may impact on the conduct of the study will require formal modification and approval by the HREBA.CC prior to implementation, and will be described transparently in subsequent reports. The study will be performed according to the Declaration of Helsinki. The study was registered at ClinicalTrials.gov before recruitment of the first participant (NCT03049384, first posted in February, 2017). The research will be conducted at a single site (Faculty of Kinesiology, University of Calgary, Alberta, Canada). This study protocol is written in accordance with the SPIRIT guidelines [63] (SPIRIT Checklist provided in Additional file 1).

### Study population

The majority of research in exercise and cancer has been conducted in female breast cancer survivors due to high prevalence and survival estimates [10]. However, CRF is not unique to a specific tumour group or cancer-treatment type. Furthermore, demographic and clinical characteristics do not moderate exercise-induced improvements in other patient-reported outcomes such as physical function and HRQL [64]. Our intention is to offer this program as widely as possible to a heterogeneous group of cancer survivors who are experiencing CRF after treatment. Therefore, male and female cancer survivors aged 18–75, following any cancer diagnosis and cancer-treatment type, will be recruited for the study. Potential participants will be screened for study inclusion using a score of CRF severity using the FACIT-F questionnaire [4] (where a score of ≤34 is the recommended cut-off point for the diagnosis of CRF [65]). Additional inclusion and exclusion criteria are presented in Table 1.

**Table 1:** Inclusion and Exclusion Criteria

Inclusion Criteria	Aged ≥ 18 and ≤ 75 years
	FACIT-F score ≤ 34
	Completion of primary treatment in ≥ 3 months and ≤ 5 years from enrolment
	Approval received from CSEP-CEP and/or physician
	Command of the English language and ability to understand instructions related to the study procedures
Exclusion Criteria	Contraindication to experimental procedures and/or exercise
	Previously diagnosed as having sleep apnea or anemia
	Currently participating in a structured exercise program or another clinical trial
	Participant is pregnant

*CSEP-CEP* Canadian Society for Exercise Physiology Certified Exercise Physiologist, *FACIT-F* Functional Assessment of Chronic Illness Therapy-Fatigue, *TMS* transcranial magnetic stimulation

### Sample size

An a priori sample size estimation was performed using G*Power 3 (v3.1.2–3.1.9 [66]). The hypothesis was treated as a time × treatment (within-between) interaction for the primary outcome measure of score on the FACIT-F. A standardized mean difference of 0.44 was used, as reported in a 2012 Cochrane review for comparisons of CRF following an exercise intervention or control, from a total of 539 cancer survivors after anti-cancer therapy [29]. Using an α level of 0.05, a 1 - β of 0.8, the total sample size was calculated as 44. Potential loss to attrition was estimated as 15%, based on previous reports from supervised exercise interventions in cancer survivors post-treatment (e.g. [67]), and thus the total sample size was calculated as 52.

### Recruitment

Participants will primarily be recruited via the Alberta Cancer Registry (Alberta Health Services, Canada). Data extraction criteria include age (≥ 18 and ≤ 75 years), diagnosed with any invasive cancer from 2013 to 2016, and postal codes within 20 km of the University of Calgary. From the resulting extraction, equal numbers of males and females will be randomly sampled and sent a letter confidentially and anonymously via the Registry. Those contacted will be under no obligation to respond to the research team. Participants will also be recruited via liaising with clinicians and/or advertising at cancer centres local to the University of Calgary. When interested participants contact the study co-ordinator (via phone or email), they will be informed about the main aspects of the research and asked about the time since their last cancer treatment, and fatigue severity. If potentially eligible, participants will be provided with the participant information sheet and encouraged to ask questions about the risks and benefits of participation. Once the participant has had time to review the information, the first visit to the laboratory (Lab Visit #1) will be scheduled.

### Randomization

Due to a continuous enrolment strategy and relatively small sample size, participants will be allocated to groups using a dynamic allocation procedure (minimization, performed using an open-source, online minimization program (MinimPy Program 0.3) [68]. In this process, the first participant is allocated randomly to one of two treatment groups. Each newly enrolled participant is hypothetically allocated to each treatment group and an imbalance score is calculated given unweighted baseline categorical variables: sex (male, female), age group (18–39, 40–49, 50–59, 60–75) and cancer type (breast, prostate, lung, colorectal, other). The participant will be allocated to the preferred group (least imbalance) with a 1:1 allocation ratio, and a randomisation weighting of 0.75 in order to avoid the potential introduction of bias associated with pure minimization [69]. Participants will be randomized following completion of all baseline assessments (i.e. after Lab Visit #2, see below), by a researcher who is independent of the recruitment, enrolment and laboratory assessment process, and who has password-protected access to the allocation procedure for the trial within the online minimization program.

### Blinding

During the assessments before the intervention, the participants and study team will be blinded to group assignment as this occurs before randomization. Following randomization, blinding of participants and the study team is not possible due to the recognised complexity of blinding to an exercise intervention [29], particularly in the case of interventions that are tailored to the individual, and where the study team will communicate about participant wellbeing. Data files will be anonymized using a code by an independent researcher who is unrelated to the study prior to processing by study personnel. Participants will be informed that there is equal possibility that they will be assigned to one of the two treatment groups, and that the relative impact of two exercise interventions is under investigation.

### Laboratory assessments

The study team and/or assessors for specific methods are extensively trained in those methods, and a number of pilot assessments were performed prior to the study. Regular internal inspections of the methods described below are carried out to maintain high methodological quality. Participants will be required to attend an exercise physiology laboratory (Faculty of Kinesiology, University of Calgary, Alberta, Canada) on four occasions. Lab Visits #1 and #2 take place before the 12-week exercise intervention, and Lab Visits #3 and #4 take place after the intervention. All laboratory visits will commence between 8 am and 9 am and will last 2.5–3 h. Participants will be advised to consume breakfast 1.5 h prior to arrival at the laboratory. Participants will be instructed to arrive to the laboratory hydrated and to refrain from alcohol, caffeine and strenuous activity for the preceding 24 h. Overall study time points are presented in Table 2.

**Table 2:** Study Time Points

Process, Outcome or Test	Time Point									
	Recruitment	Pre-Intervention		Intervention			Post-Intervention		Follow-Up	
		Lab Visit		Week (Total = 12)			Lab Visit		Month	
		#1	#2	3	6	9	#3	#4	6	12
Informed Consent		×^a^								
PAR-Q+		×^a^								
ECG		×^a^								
Blood Pressure		×^a^					×			
Resting HR		×^a^					×			
Participant Information		×								
FACIT-F	×		×	×	×	×		×	×	×
ERAS-r			×	×	×	×		×	×	×
FACT-G			×					×		
CES-D		×			×		×	×		
BPI-sf		×			×		×			
SPS		×					×			
GLTEQ		×					×		×	×
ISI		×					×			
Venous Blood Sample		×					×			
Body Mass		×	×				×	×		
Stature		×					×			
Neuromuscular Familiarization		×								
Start of 15-day Actigraphy		×						×		
Sleep Diary		×						×		
HRV			×					×		
Maximal Exercise Test		×					×			
DXA			×					×		
CSA			×					×		
Grip Strength			×					×		
Neuromuscular Function			×					×		
Intermittent Cycling Test			×					×		

^a^Indicates that the item is part of the screening process. BPI-sf, Brief Pain Inventory Short Form, CES-D, Center for Epidemiological Studies on Depression Scale; CSA, cross-sectional area; DXA, dual-energy X-ray absorptiometry; ECG, electrocardiogram; ESAS-r, Edmonton Symptom Assessment System (revised version); FACIT-F, Functional Assessment of Chronic Illness Therapy Fatigue Scale; Functional Assessment of Cancer Therapy - General (FACT-G); GLTEQ, Godin Leisure-Time Exercise Questionnaire; HR, heart rate; HRV, heart rate variability; ISI, insomnia severity index; PAR-Q+, Physical Activity Readiness Questionnaire for Everyone; SPS, Social Provision Scale

### Lab visit #1

#### Informed consent

Following initial communication about the main aspects of the research during the recruitment process, Lab Visit #1 will begin with an in-person discussion with the study coordinator (20–30 min). The participant will have the opportunity to express any concerns and/or ask questions. The participant information sheet will be reviewed and/or explained such that the information is comprehensible, and the study coordinator will seek verbal assurance that the participant understands the research, and that their participation is entirely voluntary. The study co-ordinator will then obtain written informed consent to participate.

#### Screening and participant information

Participants will complete a Physical Activity Readiness Questionnaire for Everyone (PAR-Q+) and will be screened for contraindications to TMS [70]. Participants will be also screened for arrhythmia and hypertension, determined during resting electrocardiography and blood pressure measurements, respectively. Continuation with the study is conditional on the screening process, and physician approval may be sought at this stage. If the participant displays a normal sinus rhythm and systolic and diastolic blood pressure of ≤144 and ≤ 94 mmHg, respectively, is cleared for physical activity by a Canadian Society for Exercise Physiology Certified Exercise Physiologist (CSEP-CEP), and no further concerns are raised that would warrant physician approval, the participant will continue to the procedures described for below. Demographics will be self-identified using a participant questionnaire, and will include sex, age, race, marital status, education, employment status and household income. Clinical variables will include cancer diagnosis, time since diagnosis, time since completion of primary treatment, treatment type (e.g. surgery, radiation therapy, hormone therapy and/or chemotherapy) and persisting side effects. Participants will self-identify as non-smokers or smokers (daily or occasional). The Alcohol Use Disorders Identification Test (AUDIT) will be used as an indication of hazardous and harmful alcohol use (score > 8) [71].

#### Participant-reported outcomes

In addition to CRF severity (Lab Visit #2), patient-reported outcomes include HRQL, depressive symptomatology, pain, social provisions, leisure-time exercise and insomnia severity. The following questionnaires will be completed in the order described and were chosen for their established reliability and validity with specific emphasis on use in cancer populations. HRQL will be assessed using the Functional Assessment of Cancer Therapy – General (FACT-G) [72], which provides scores for subscales of physical well-being, social/family well-being, emotional well-being, functional well-being, and additional concerns specific to cancer type. In addition, the sum of these subscale scores will be used to calculate total score for HRQL. Depressive symptomatology will be assessed using the 20-item Center for Epidemiological Studies on Depression Scale (CES-D) [73]. Pain will be evaluated using the Brief Pain Inventory Short Form (BPI-sf) [74] which measures both pain severity and the impact of pain on functioning (interference). Social provisions will be assessed using the Social Provision Scale (SPS) [75], which provides scores for six sub-groups: guidance, reliable alliance, reassurance of worth, attachment, social integration, and opportunity for nurturance. A total SPS score will be derived from the six sub-scales. Leisure-time exercise will be assessed using a modified Godin Leisure-Time Exercise Questionnaire (GLTEQ) [76], including the frequency and duration of mild, moderate, strenuous, resistance and flexibility exercise.

#### Venous blood sample

A venous blood sample (total volume 35 mL) will be collected from the antecubital fossa by a certified phlebotomist, between 9:30–10:30 am (≥ 2 h post-prandial). The sample will be analyzed for whole blood count and variables including catecholamines, serotonin, cortisol, and cytokines including tumor necrosis factor-α, interferon-γ, transforming growth factors (TGF-β1, 2 and 3), interleukins (IL-1β, 2, 4, 5, 6, 8, 10, 12 and 13), monocyte chemoattractant protein-1 and granulocyte-macrophage colony-stimulating factor. Whole blood count will be analyzed within 2 h of collection at the laboratory of Foothills Medical Centre. Other parameters assessed from blood serum and plasma will be centrifuged at 4 °C and 3000×g for 15 min, divided into aliquots and stored at − 80 °C. Samples will be stored until laboratory evaluation for the current study only, and will not be stored for use in any ancillary or future study.

#### Maximal exercise test

Following measurement of stature (cm) and body mass (kg), a maximal exercise test will be conducted for the measurement of maximal oxygen uptake ($\dot{V}{\mathrm{O}}_{2\max }$). The test will be conducted on a custom-built recumbent ergometer, which uses an electromagnetically-braked Velotron system (RacerMate Inc., Seattle, WA). Seat position will be adjusted for each participant, and self-selected cadence will be determined (≥ 60 rpm). These details will be recorded for replication in subsequent visits. Participants will be instrumented for the measurement of heart rate (HR) and breath-by-breath pulmonary gas exchange and ventilation (Quark CPET, COSMED, Rome, Italy). The starting power output (25–50 W) and increment (10–20 W) will be adjusted on an individual basis as that which is estimated to result in a test of 8–12 min duration. The power will be increased at 1-min intervals until volitional exhaustion. Rating of perceived exertion (RPE) (Borg’s RPE scale [77], administered according to published instructions [78]) and dyspnea (Borg CR-10 scale [79]) will be recorded every minute. For all cycling tests, verbal encouragement will be provided by the same experimenters every 20–60 s [80]. A fingertip blood sample will be collected at exercise cessation for the analysis of blood lactate (Lactate Scout^+^, EFK Diagnostics, Cardiff, UK) and participants will complete a supervised cool down. The highest 30-s average oxygen uptake will be calculated and a plateau in oxygen uptake will be verified according to published criteria [81, 82]. Where not achieved, the measure will be referred to as peak oxygen uptake ($\dot{V}{\mathrm{O}}_{2\mathrm{peak}}$).

#### Neuromuscular familiarization

Participants will complete a 45–60 min familiarization to the neuromuscular assessments described in detail under Lab Visit #2.

#### Actigraphy

Participants will be provided with a MotionWatch 8© actigraphy system (CamNtech, UK) and instructions for use in the study. This is an unobtrusive, waterproof, wrist-worn device containing a light sensor and a tri-axial accelerometer detecting acceleration in a 0.01–8 g range. The device will record activity counts and light intensity in 30-s epochs, as recommended by the manufacturer. The device will be worn on the non-dominant wrist for a continuous 15-day period, in accordance with established recommendations [83, 84]. Participants will also be provided with a sleep diary to complete alongside the actigraphy measurement [85].

#### Heart rate variability

Short-term heart rate variability (HRV) will be measured before and after the intervention. Participants will receive a HR monitor (S810i Polar, Polar Electro, Kempele, Finland; sampling rate 1000 Hz) to complete a 10-min HRV measurement in their own home. To control for transient variables [86], the participant will be instructed to follow a normal sleep routine the night before the measurement, avoid strenuous physical activity and alcohol for the preceding 24 h, complete the measurement immediately upon waking (after visiting the washroom if necessary) and thus prior to any food or caffeine intake, in the supine position, under quiet and thermoneutral conditions.

### Lab visit #2

Participants will return to the lab after completion of the 15-day actigraphy measurement. Participants will return the actigraph and sleep diary and complete the below assessments for Lab Visit #2.

#### Cancer-related fatigue

Score on the FACIT-F will be used to assess CRF severity (primary outcome). The revised Edmonton Symptom Assessment System (ESAS-r) tiredness scale will also be used [87, 88], as recommended for the screening of CRF severity in the clinical practice guidelines for standard cancer care in Alberta [89]. The questionnaires will be completed between 8:30–9:30 am.

#### Body composition and bone mineral density

Participants will undergo a whole-body scan using dual energy X-ray absorptiometry (DXA; Discovery W, Hologic, Bedford, MA), for the assessment of parameters relating to body-composition and bone mineral density.

#### Muscle cross-sectional area

Cross-sectional area (CSA) of the right vastus lateralis (VL) and rectus femoris (RF) will be assessed using real-time B-mode ultrasonography (M2540A, Phillips, Bothell, WA). Participants will adopt a supine position with legs relaxed and knees extended. One axial perpendicular line will be marked with indelible ink at 50% of the distance between the greater trochanter and the lateral epicondyle of the knee. After 20 min of rest, sufficient water-soluble gel will be applied to the transducer to ensure that clear images are obtained with minimal and consistent pressure to avoid compression of the muscle during examination. Participants will be asked to fully relax the muscle while consecutive two-dimensional (2-D) images are acquired with the probe placed perpendicular to the skin at the anatomical site. Three images will be acquired by the same experimenter. CSA will be estimated through manual tracing of the muscle borders using open-source imaging program (ImageJ).

#### Grip strength

The grip bar of a handgrip dynamometer will be adjusted for each participant and recorded for post-intervention measurement. The measurement will be made in a standing position, with the elbow extended and arm parallel to, but not touching, the side of the body. Grip strength (kg) for the dominant hand [90] will be assessed as the highest of three ~ 3-s maximal efforts, separated by 1-min rest.

#### Neuromuscular assessments

**Fig. 2:**
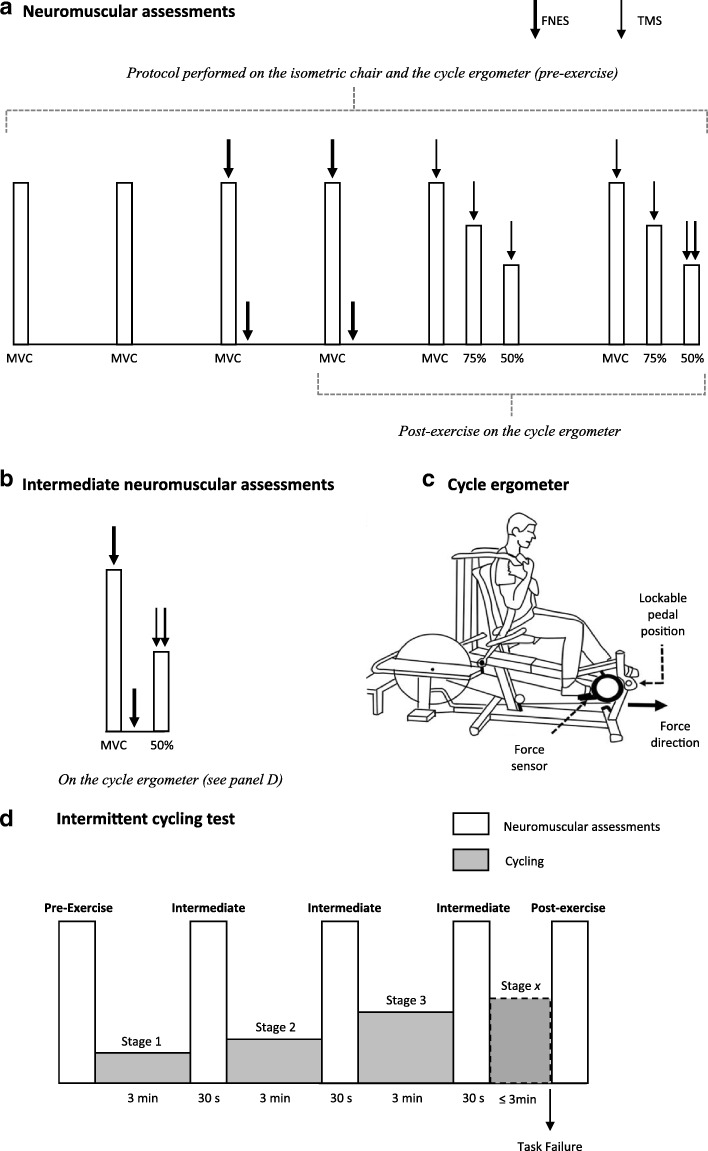
A schematic illustrating: (panel **a**) the neuromuscular assessment performed pre- and post-exercise; (panel **b**) the intermediate neuromuscular assessments performed every 3 min as part of the intermittent cycling protocol; (panel **c**) the cycle ergometer; and (panel **d**) the intermittent cycling protocol including neuromuscular assessments. MVC, maximal voluntary contraction; 75 and 50%, the percentage of the preceding MVC; TMS, transcranial magnetic stimulation; FNES, femoral nerve electrical stimulation

All neuromuscular data will be acquired using a PowerLab 16/35 and LabChart v8 software (ADInstruments, Bella Vista, Australia), and further measurement details are described in later sections. For neuromuscular assessments, three preliminary procedures will take place on an isometric chair as: (i) determination of a supramaximal femoral nerve electrical stimulation (FNES) intensity; (ii) determination of optimal coil position for TMS; (iii) determination of optimal stimulation intensity for TMS. On the isometric chair, participants will also perform a set of preparatory contractions of 5-s duration, with 5 s rest between contractions. The preparatory contractions involve five at 10%, five at 30%, three at 50% and two at 75% of a familiarization MVC. Participants will then perform a neuromuscular assessment (Fig. 2a), starting with two MVCs with no stimulations, separated by 60 s. Where the two MVCs differ by ≥5%, a third will be performed. All MVCs (duration 3–5 s) will be performed with strong verbal encouragement and visual feedback of force displayed on a large computer monitor positioned ~ 1 m in front of the participant. Next, two MVCs with single FNES delivered during the plateau of the MVC, and within 2 s of relaxation will be performed (for the calculation of voluntary activation with FNES [VA_FNES_], also see *Data Analysis*). Finally, participants will perform two sets of contractions at 100, 75 and 50% MVC, separated by 5-s rest, with 20-s rest between sets (for the calculation of voluntary activation with TMS [VA_TMS_], see also *Data Analysis*). Guidelines at 75 and 50% of the preceding MVC for each set will be plotted without any delay using a custom-made macroinstruction. TMS will be delivered during each contraction when force has plateaued or stabilized on the target guideline. Participants will be instructed to resume the contraction immediately after TMS delivery (in order to better evaluate silent periods, see also *Data Analysis*). During the voluntary contraction at 50% MVC in the second set, FNES will also be delivered after the TMS, once the participant has returned to the guideline. The participant will be transferred to the cycle ergometer shown in Fig. 2c, to repeat this neuromuscular assessment before cycling exercise (Fig. 2a).

#### Incremental cycling test and neuromuscular fatigue

Following the pre-exercise assessment on the ergometer, participants will complete an incremental cycling test (Fig. 2d). Immediately post-exercise i.e. within 1 s from task disengagement (or rpm < 60), a post-exercise neuromuscular assessment will be performed (Fig. 2a). The incremental protocol involves stages of 3-min duration. Between each 3-min stage, the pedals will be locked, and an intermediate assessment will be performed (Fig. 2b). As shown in Fig. 2b, this involves an MVC with FNES, and a contraction at 50% MVC with TMS and FNES. After the intermediate assessment (30 s duration), the pedals will be unlocked and the participant will resume cycling at their target cadence at the pre-determined higher power output for the next stage. Increments in power output are 0.3 W·kg^− 1^ for the first four stages, 0.4 W·kg^− 1^ for the following five stages and by 0.5 W·kg^− 1^ for any subsequent stages. Cadence will be the only real-time feedback participants will receive during cycling, and verbal instructions will be given should cadence drift by ≥4 rpm. The reliability of this incremental protocol has previously been assessed in our laboratory in healthy individuals [61] and cancer survivors (unpublished data). The procedures described are covered in detail during the neuromuscular familiarization of Lab Visit #1.

#### Measurement of voluntary and evoked force

On the isometric chair (custom made from a Kin-Com dynamometer frame), force will be measured during voluntary and evoked contractions using a calibrated load cell (LC101-2 K, Omegadyne, Sunbury, OH) connected to a noncompliant cuff attached around the right ankle, just superior to the malleoli. The load cell will be adjusted to position it directly behind the point of applied force. Participants will sit upright with the knees and hips at 90° flexion, secured using straps across trunk and shoulders. On the ergometer, force will be measured during voluntary and evoked contractions using a wireless pedal force analysis system located between the pedal and crank (PowerForce Model PF1.0.0, Radlabor GmbH, Freiburg, Germany). The ergometer permits the pedals to be locked instantly in a fixed position with hip angle at ~ 100° and right knee and at ~ 90°, and the crank parallel to the ground [61]. This allows participants to perform a contraction whereby force is measured in line with the crank. Participants will be secured with non-compliant straps across the trunk. Force will be sampled at 500 Hz and recorded using Imago Record (version 8.50, Radlabor GmbH). To provide real-time visual force feedback, the PowerForce signal will be transmitted to the PowerLab system using a National Instruments 16-bit A/D card (NI PCI-6229, National Instruments, Austin, TX) and connector block (BNC-2111, National Instruments).

#### Measurement of Electromyographic responses

Surface electromyography (EMG) will be recorded from the right vastus lateralis (VL) and rectus femoris (RF), and the long head of the biceps femoris (BF). The skin will be shaved, abraded and cleaned with isopropyl alcohol wipe to ensure a low impedance (< 10 kΩ). Two single-use electrodes (10-mm diameter, Meditrace 100, Covidien, Mansfield, USA) will be placed in a bipolar configuration (inter-electrode distance of 20 mm) over the muscle belly following SENIAM recommendations [91]. The reference electrode will be placed over the patella. Raw EMG signal will be analog-to-digitally converted, amplified (octal bio-amplifier ML138, ADInstruments; common mode rejection ratio = 85 dB, gain = 500) and sampled at 2000 Hz. EMG will be band-pass filtered (5–500 Hz).

#### Femoral nerve electrical stimulation

Electrical stimuli (1 ms pulse width) will be delivered using a constant-current stimulator (DS7AH, Digitimer Ltd., Hertfordshire, UK). The cathode (10-mm diameter, Meditrace 100) will be positioned over the femoral nerve, high in the femoral triangle. The electrode will be secured with tape and a gauze plug to apply pressure. The anode (50 × 90 mm, Durastick Plus, DJO Global, Vista, CA) will be placed midway between the greater trochanter and the iliac crest. For the determination of supramaximal FNES intensity, single FNES will be delivered beginning at 10 mA and increasing by 10 mA until no further increase in twitch force or VL M-wave amplitude can be elicited. The intensity at this plateau will then be increased by 30%.

#### Transcranial magnetic stimulation

Single TMS pulses (1-ms duration) will be delivered with a 110-mm diameter concave double-cone coil powered by a mono-pulse magnetic stimulator (Magstim 200^2^, The Magstim Company Ltd., Whitland, UK) with the coil orientated to induce a postero-anterior intracranial current flow. Optimal coil position will be defined as the location eliciting the largest motor evoked potential (MEP) in the VL and RF and a concurrent small MEP in the BF with stimulations delivered at 50% maximal stimulator output (MSO) during brief contractions at 20% MVC, with 10 s rest between contractions. A standardised procedure will be used involving six potential sites marked on a white Lycra swim cap worn by participants. The six sites (A – F) include the vertex (A), 1 cm (B) and 2 cm (C) posterior to the vertex along the nasion-inion line, 1 cm lateral to vertex over the left motor cortex (D), and 1 cm (E) and 2 cm (F) posterior to D. When optimal stimulation site is selected and marked clearly on the swim cap, the stimulation intensity will be determined using a standardised procedure involving stimulations at 50, 60, 70 and 80% MSO (randomized order, four stimulations at each intensity) delivered during brief contractions to 20% MVC [92], with 10 s rest between contractions. The intensity will be optimised for the measurement of VA_TMS_. That is, the intensity eliciting a maximal VL and RF MEP will be selected (i.e. the lowest intensity resulting in an increase of less than 5% MEP amplitude at higher stimulus intensities). The size of the superimposed twitch (SIT) will also be examined to ensure that this intensity corresponds to a maximal SIT at 20% MVC (as the SIT can be lower at higher TMS intensities due to co-activation of the knee flexors).

### Post-intervention assessments

After the intervention, Lab Visit #3 will be completed 72–96 h after the final exercise session. Lab Visit #3 involves the post-intervention assessment of patient reported outcomes, a venous blood sample, maximal exercise test (replicated starting power output and increment) and (re)familiarization to neuromuscular measures as described for Lab Visit #1 (see also Table 2). Lab Visit #4 will be completed 72–96 h after Lab Visit #3. Lab Visit #4 assessments are identical to those described for the pre-intervention Lab Visit #2, including replication of power outputs during the incremental cycling test (i.e. power outputs will be the same absolute value, even where body mass has changed). The sleep diary and actigraph measurement will begin after Lab Visit #4, and collected from participants after the 15-day measurement period.

### Follow-up

Six and 12 months after the exercise interventions, participants will be contacted via phone or email to complete the FACT-F scale, ESAS-r tiredness scale and the GLTEQ.

### Treatment arms

Both exercise interventions will take place in the Thrive Centre, a fitness facility for people affected by cancer (Faculty of Kinesiology, University of Calgary, Alberta, Canada). The exercise intervention will be delivered in a small group setting by exercise specialists, who have completed specific cancer and exercise training (http://thrivehealthservices.com). Participants in both treatment arms will be supervised by the same exercise specialists. The following prescriptions for aerobic and/or resistance exercise will be followed only where achievable, such as where the exercise is not voluntarily terminated prematurely due to intolerable levels of perceived fatigue, dyspnea or muscle weakness. Where an existing adverse effect of treatment (e.g. shoulder dysfunction) or injury (e.g. knee replacement) limits the performance of a movement, the movement will be restricted, modified or substituted to ensure that there is no pain during or after exercise. In addition, the exercise specialist may adjust intensity or duration based on observation and judgement, particularly in the case of reducing the demands to accommodate day-to-day fluctuations in health or wellbeing in a diverse group of cancer survivors. A decision to discontinue an individual’s intervention will be made by the research team if there is concern that the exercise intervention is causing harm. Upon arrival to supervised exercise sessions (i.e. before exercise), participants will be asked to indicate their fatigue levels using a rating-of-fatigue scale, which quantifies the intensity of the subjective feeling state at a given moment [93]. Should the exercise intervention appear to gradually increase the rating of fatigue over 1–2 weeks, the intensity will be reduced as deemed necessary to ensure the overall wellbeing of the participant. The exercise specialist and research team will communicate regularly regarding individual participants. Participant safety is paramount and any adverse events (related to exercise or not) will be monitored and reported according to the standardized guidelines for reportable events from the independent ethics committee (HREBA.CC). The reasons for dropout from the intervention will be recorded where possible, and no further outcome data will be collected in participants who withdraw from the study.

In both treatment arms (see *Traditional Exercise Group* and *Tailored Exercise Group*, below), each supervised exercise session will include a low-intensity warm-up (5 min light cycling and 5 min dynamic stretching or mobility exercises), and a cool-down which will include stretching of major muscle groups. Adherence to the exercise intervention will be reported as the number of sessions attended as a percentage of total sessions scheduled (with a maximum of 36). Where the participant has a commitment known in advance such as a medical appointment or holiday, the missed sessions will be rescheduled or replaced at the end of the 12-week intervention. Unanticipated cancellations (non-attendance) will not be substituted. Exercise sessions will primarily be offered on weekday afternoons/evenings with flexibility to ensure that people who have returned to work and/or are caring for dependents are logistically able to participate.

Participants in both treatment arms will be provided with an identical intervention booklet during the first training session. The booklet contains weekly physical activity logs and guidelines for ~ 10 static stretches. The physical activity logs will be used as a self-report measure of additional physical activity (e.g. a brisk walk) that is undertaken outside of the exercise intervention (such additional physical activity is not restricted/prohibited). The booklet also contains educational information related to promoting participant adherence, retention, and long-term behavioural change [94, 95]. The booklet is written in a plain language, and includes sections on goal setting, planning for barriers, monitoring behaviour, maintaining motivation and enhancing personal control. The information in the booklet will be discussed verbally by the supervising exercise specialist, to ensure comprehension and to encourage engagement with the material. When the study is complete, participants will be encouraged to continue attending the Thrive Centre as a ‘drop-in’, during regular scheduled time-slots when the facility is monitored by volunteers with specific cancer and exercise training.

#### Traditional exercise group

Participants in the traditional exercise group will engage in exercise of a duration, frequency and intensity that is consistent with published recommendations and clinical practice guidelines for cancer survivors (e.g. [33–36]), and as such, compatible with health-related physical activity guidelines for the general population. The goal of the intervention is to progress to meet guidelines of 150 min per week of moderate-intensity aerobic exercise, and resistance training on at least two days per week. Aerobic exercise will be performed on a stationary cycle ergometer, rowing ergometer, treadmill and/or elliptical trainer (participant’s preference). The aerobic exercise duration will be progressive such that in weeks 1–4, exercise will be performed for 30 min on three supervised sessions per week. The total aerobic exercise duration will progress from 90 min in weeks 1–4, 120 min in weeks 5–8 and 150 min in weeks 9–12 over 3 sessions per week. The intensity of exercise will correspond to an RPE of 11–14, which is in line with published guidelines [35, 96] and empirical data [97] for moderate exercise. Participants will have been familiarized with the RPE scale and instructions on both Lab Visits #1 and #2. The corresponding HR and equipment resistance/speed will be monitored and recorded during every session.

Two sessions per week (separated by ≥48 h) will include resistance training after the aerobic component, involving exercises targeting all major muscle groups. Participants will perform one to three sets of eight to twelve repetitions. Within these guidelines, the principle of progressive overload will be applied to gradually increase training volume. There will be a 1–3 min rest period between sets, and contractions will be performed at slow to moderate velocities [98]. Eight to ten body mass/dumbbell exercises will be selected in 3-week micro-cycles from a pre-determined bank of ~ 30 exercises selected by the research team. Appropriate individual modifications and progressions from a novice level will be included. Participants will be coached in correct technique for each movement. Exercises will be prescribed with consideration of an individual’s cancer or cancer-treatment side effects (e.g. lymphedema, peripheral neuropathy), and awareness of increased risks based on cancer or cancer-treatment (e.g. bone fracture in those with previous bone metastases). Specific guidelines in this regard will be followed where available (e.g. [33]).

#### Tailored exercise group

The experimental tailored exercise group will be prescribed an intervention designed specifically to address the deficits or areas for improvement identified in Lab Visits #1 and #2. The optimisation of the exercise intervention is based on the outcome of interest i.e. CRF. As the mechanisms of CRF are unknown, we have chosen to focus primarily on tailoring the intervention to improve specific physiological parameters and/or sleep, with consideration of the whole profile of baseline assessments. The results of an individual’s assessment will be reviewed and discussed by the research team and exercise specialists to optimize the intervention. For transparency and to assist with interpretation of generated data where the interventions vary between participants, the characteristics of the individual (anonymized) tailored exercise interventions (e.g. the frequency, intensity, duration, RPE, HR and/or the type of movement) will be made available in an open-access repository upon completion of the study. The design and application of this experimental exercise intervention will proceed with attention to the principles of exercise training and be evidence-based [99]. The frequency (three times per week) and total duration (60–90 min) of sessions (and therefore contact and interaction with exercise instructors and other participants) are the only aspects of exercise dose that are designed to be equivalent to the traditional exercise group. The intervention will be adjusted at 3-week intervals based on participant feedback and the judgement and observations of the research team/exercise specialists. Three examples of the parameters and resulting focus of tailored exercise interventions are provided below. A diverse group of cancer survivors will have diverse profiles based on Lab Visits #1 and #2, and therefore the intervention may be multi-modal i.e. involve a combination of the examples provided below.

(i) Based on a low muscular strength (force-generating capacity in the knee extensors) at baseline (in comparison to non-fatigued cancer survivors and healthy adults of the same sex and similar age; data collected in our laboratory), the exercise intervention will focus on improving this using both neuromuscular electrical stimulation (NMES) and resistance training with voluntary contractions. For the former, NMES is widely applied to the quadriceps as a (re)training modality, including in pathological conditions where muscle weakness is an issue [100, 101]. In terms of resistance training, if muscle mass is low (on consideration of VL and RF cross-sectional area, and in comparison to reference values for DXA-derived lean mass index [102, 103]), the focus will be on hypertrophy. High repetition multi-set resistance training will be incorporated [104], with additional focus on eccentric actions [105, 106]. Prescribed aerobic exercise will be minimal [107], particularly in the case of low body mass or history of malnutrition during treatment. Where muscle mass appears to have been maintained, concurrent to low VA, resistance training may progress (safely) to involve sets of low repetitions and high loads [104].
(ii) Based on substantial cardiorespiratory deconditioning, primarily based on a low $\dot{V}{\mathrm{O}}_{2\max }$ according to age-group norms [96], participants will be prescribed interval training on at least two (of three sessions) per week. Supervised high intensity interval training (HIIT) results in improvements in cardiorespiratory fitness and other outcomes in cancer survivors, and can be considered low risk in regards to adverse events [108]. The evidence of safety (in regards to the low risk of cardiovascular events in particular) has been convincingly demonstrated in other clinical populations e.g. coronary heart disease patients [109]. HIIT will be performed on a cycle ergometer to reduce risk of muscular-skeletal injury. Participants will be familiarised with HIIT gradually, and the intensity of the work intervals will be increased over the first two weeks depending on tolerance, to reach 85–95% of peak HR. Due to the relatively recent adoption of HIIT in cancer populations, there are no guidelines on optimal HIIT prescription (e.g. work:rest ratio and interval duration), where the effectiveness of different HIIT protocols should be tailored to ensure it is feasible for the individual. However, the recommendations from other clinical populations will be implemented such that the typical work:rest ratio will increase to ≥1 and work intervals ranging from 30 s up to 4 min. For example, the 4 × 4 protocol [110] (4 × 4 min work with 3 min active recovery at the lowest possible intensity) will be appropriate for many cancer survivors.
(iii) Based on substantial sleep disturbance determined via actigraphy (e.g. those who display three of the following criteria: total sleep time ≤ 440 min; sleep efficiency [total sleep time as a percentage of time in bed] ≤ 87%; sleep onset latency [an index of the difficulty in the transition from wake to sleep] > 14 min; wake after sleep onset ≥25 min [111, 112]), there will be a focus on exercise to improve sleep. The identification of sleep disturbance will be primarily based on actigraphy, but subjective complaints of sleep disturbance will also be considered (ISI score and subjective total sleep time from the sleep diary) with awareness of the misperception of sleep relative to objective measures [113]. Although exercise is a widely recognised intervention to improve disturbed sleep, there are many unanswered questions in regards to optimally prescribing exercise interventions for this purpose (e.g. dose, mode, timing) [114]. However, in adults, evidence suggests that exercise duration moderates sleep outcomes for regular exercise (where longer duration is more beneficial) [115] and most studies have used moderate aerobic exercise such as walking [116]. Overall, the evidence suggests that exercise improves sleep in cancer populations, though few studies have investigated this in cancer survivors after treatment who present with sleep disturbance at baseline (reviewed in [43]). Nevertheless, the intervention will focus on long-duration (progressing to e.g. 60 min) aerobic exercise such as walking.

A further consideration is whether the participant is at increased health risk due to obesity (body mass index > 30 kg/m^2^, with additional consideration of percentage body fat from DXA). If obese, the intervention will involve low impact activity that puts minimal stress on joints (elliptical trainer, cycling or walking) to avoid injury, with increasing duration (progressing to > 150 min per week) on intensity to increase energy expenditure and assist with weight management [117].

### Data monitoring

A data monitoring committee was not included because the trial involves a behavioural intervention (progressive exercise) with known/minimal risks, and does not require periodic benefit–risk assessments. No independent auditing of trial conduct is planned.

### Confidentiality

In order to maintain confidentiality during and after the trial, all study-related information will be stored securely at the study site in areas with limited access. Furthermore, access within the study team will be the minimum required for data analysis and quality control. Blood samples, electronic files, data sheets and completed questionnaires will be stored using coded IDs. Digital files will be stored on password-protected computers, in password protected folders, and backed up on a password-protected hard drive. Records that contain personal identifiers (such as informed consent forms) will be stored separately from those identified by coded ID, in a locked cabinet in an office accessible to the study co-ordinator.

### Data analysis

#### Neuromuscular data

The potentiated mechanical response from a single electrical stimulus will be analysed for amplitude of the twitch (Q_tw,pot_), maximal rate of force development and maximal relaxation rate. Voluntary activation using femoral nerve electrical stimulation (VA_FNES_) will be calculated using the interpolated twitch technique where the amplitude of the SIT is normalized to the corresponding Q_tw,pot_ using the equation VA_FNES_ (%) = (1-(SIT/Q_tw.pot_)) × 100 [60]. For TMS, an estimated resting twitch (ERT) will be calculated by taking the y-intercept of a linear regression of the SIT-voluntary force relationship. VA_TMS_ will be subsequently quantified using the equation VA_TMS_ (%) = (1-(SIT/ERT)) × 100 [118]. Where regressions are not linear (defined as *r* < 0.9 [119]), those data will be excluded.

For the evoked EMG responses, the peak-to-peak amplitude and area under the curve of the MEP in all muscle groups will be determined from a selection of data encompassing the biphasic wave. The selection will begin at the first deviation from zero after any stimulation artefact, and end on the return to zero after the biphasic wave. The M-waves evoked in the VL and RF will be analysed using the same method. For the assessment of corticospinal excitability, the VL and RF MEPs will be normalised to an M-wave delivered during a contraction and nearby in time_._ The silent period will be measured from stimulus artefact to the continuous resumption of voluntary EMG, determined by an experimenter experienced in the analysis, using visual inspection of the EMG trace [120].

#### Heart rate variability

In the time domain (ms), the mean normal-to-normal (NN) interval, the standard deviation of the average NN interval (SDNN) and the square root of the mean squared differences of successive NN intervals (RMSSD) will be calculated. As recommended for short-term HRV recordings [115], the spectral components analyzed in the frequency domain (ms^2^) will be the very low frequency (VLF; 0.01–0.04 Hz), low-frequency (LF; 0.04–0.15 Hz) and high-frequency (HF; 0.15–0.40 Hz). The LF/HF ratio will also be calculated. The spectral analysis will be performed using fast Fourier transform algorithms (Kubios HRV Standard v3.0.2).

#### Actigraphy

The night following Visit #1 will be excluded from the actigraphy data analysis to mitigate any effect of acute maximal exercise (which participants are likely to be unaccustomed to). Data will be analysed using MotionWare 1.0.27 (CamNtech, UK). Responses from the sleep log will be used to confirm the start and end time of the sleep window, activity onset/offset and “lights out”/“lights on” (as determined by the light sensor). Sleep parameters calculated within the software include time in bed, actual sleep time, actual wake time, sleep efficiency (the percentage of time in bed spent sleeping), sleep-onset latency (time from “lights out” to sleep onset), fragmentation index (the percentage of immobile phases of one minute). For rest-activity cycle characteristics, the following parameters will be calculated using a non-parametric circadian rhythm analysis option [121]: relative amplitude (calculated from estimated lowest and highest activity periods), inter-daily stability (the degree of regularity of the rest-activity patterns on individual days in the 24 h environment), intra-daily variability (the fragmentation of periods of rest and activity), the estimated peak time of activity period, mesor (mean level), L5 (mean activity counts in the least active 5 h period in the average 24 h pattern) and L5 mid (the central time of the L5 period, usually referring to the trough of the rest-activity cycle). The mean amount of activity during the sleep period and the activity index (percentage of 30-s epoch during both sleep and wake periods with an activity > 0) will be calculated from extracted raw data. An objective measure of day-time physical activity will be computed as the number of minutes spent at sedentary, light and moderate-to-vigorous physical activity intensities over the 14-day measurement period. This will be quantified using calibrated cut-points for MotionWatch 8© activity counts, as determined in healthy older adults [122].

### Intended statistical analysis

Data will be analysed after data collection is complete, and no interim statistical analysis will be performed. Descriptive statistics will be used for demographic and clinical variables measured at baseline for each group. Frequencies and percentages will be used for categorical variables and the mean ± standard deviation (or median and range) will be used for continuous variables. To account for any differences in loss to follow up between groups, the primary analysis will be conducted on an intention-to-treat basis. For the primary outcome of FACIT-F score between treatment arms over time, data will be analysed with linear mixed models using R [123] and lme4 [124]. “Treatment arm” will be included as a fixed effect and “participant” as a random effect. Parameters will be estimated using restricted maximal likelihood. The Kenward-Roger approximation for degrees of freedom will be used when evaluating the significance of effects. This produces optimal type I error rates (neither anti-conservative nor overly sensitive to sample size) [125]. Secondary analyses will be performed to assess adjustments for protocol deviations (per protocol analysis). Statistical code will be made openly available upon publication of the results. A minimum of a 3-point difference in FACIT-F score will be considered clinically relevant [126]. For secondary outcomes that are assessed pre- and post-intervention only (e.g. neuromuscular measures, sleep parameters, blood biomarkers), two-way mixed design ANOVA will be used (group [tailored vs. traditional] × time [pre- vs. post-intervention]). In this case, missing data will be dealt with using list-wise deletion. Following a significant interaction, pairwise comparisons will be conducted with a Bonferroni adjustment. The threshold for rejecting the null hypothesis will be *p* < 0.05. For main and interaction effects, partial eta squared will be computed as an effect size estimate. Effect sizes for pairwise comparisons will be reported as Cohen’s *d* [127, 128]. This will be supplemented with 95% confidence intervals for mean differences. Further exploratory analysis will be labelled as such in later reporting.

### Dissemination and data sharing policy

The results for primary and secondary outcomes will be disseminated regardless of the magnitude or direction of the effect. The primary research aim will be addressed in a main publication reporting the results of the primary analysis i.e. the effect of treatment arm on CRF severity. Due to the number of secondary outcomes, additional publications may be warranted to provide in-depth analysis of, for example, data related to neuromuscular fatigue, sleep or ROF. Scientific and administrative information about the results of the trial will be submitted to the ClinicalTrials.gov results database. Participants will be informed about their personal results in a participant report written in plain language, within 4 weeks of Visit #4. No later than 2 years after the final follow-up assessment, an anonymized, de-identified dataset will be made openly available to an appropriate data archive for sharing purposes.

### Discussion

To the best of our knowledge, this will be the first study to examine and compare the effects of a traditional exercise intervention against a tailored exercise intervention on CRF in cancer survivors. Furthermore, this will be the first study to include a comprehensive examination of potential pathways for the improvement in CRF with exercise, including patient-reported outcomes such as depressive symptomology and pain severity, alongside objective assessments of blood biomarkers, physical activity levels, sleep and cardiorespiratory fitness. In addition, we will examine the neuromuscular correlates of CRF, including neuromuscular function at rest and neuromuscular fatigue during an exercise task that is dynamic and involves large muscle groups i.e. relevant to daily activities such as locomotion.

The most important decision about the design of this study also represents the most significant challenge. That is, tailored exercise interventions that are designed based on comprehensive (though not exhaustive) pre-intervention assessments. It is not possible to specify all possible aspects of this tailoring in advance, though a number of categories have been detailed. The basis of the tailored intervention is that it involves consideration of individual profiles. For most cancer survivors, the published guidelines offer a foundation for exercise recommendations, given that exercise is considered as being low risk with large potential for benefits. However, in clinical practice and during treatment, exercise prescription does involve tailoring based on adverse events or as recently highlighted, co-morbidities [38]. We propose that specific tailoring may also be necessary for a diverse group of cancer survivors with persistent CRF after treatment completion. Targeting CRF as a symptom, rather than a tumor group or treatment type, will undoubtedly result in a heterogeneous cohort. Although the primary research question is clearly defined, there are multiple degrees of complexity for later interpretation and reporting of study results. Our solution is to make the (anonymized, de-identified) intervention data openly accessible, with the restriction that participant privacy/confidentiality must be maintained. To our knowledge, this is not common practice in exercise oncology research, but will facilitate replication of the characteristics of individual interventions and allow further exploratory analysis in regards to the interpretation and comparison of intervention data.

As this is a single-site study, participants must be able to regularly travel to the University of Calgary, such that it is only feasible for cancer survivors who live locally to participate. We recognise that this is a limitation of the study. In terms of reducing barriers to recruitment for people who live locally, parking costs at the site will be reimbursed. However, participants must also have the time available to participate in an exercise intervention. As the time post-treatment is up to five years, it is anticipated that a number of potential participants will be struggling with CRF after having returned to work and/or while also caring for dependents. To help with overcoming this barrier, exercise sessions will be offered with a large degree of flexibility in regards to the day of the week and the exercise time, which will also be an important factor in regards to participant retention.

In this study, the traditional exercise group was designed as an active control, and considered to be an appropriate comparator to the (experimental) tailored exercise group. A ‘no exercise’ wait-list control was not included in the study design due to the established benefit of exercise on CRF [29–31]. We did not consider it necessary to confirm that an improvement in CRF with exercise was superior to an improvement due to, for example, an additional 3–4 months in the passage of time since cancer treatment. Due to the difficulties in blinding, the active control has been designed to not only be broadly consistent with published guidelines, but to be equivalent to the experimental group on non-specific conditions (that may influence the primary outcome, which is a perceptual construct) such as expectancy, social support during exercise or attention from the exercise specialist [129]. The continuous enrolment strategy is due to the anticipated difficulty in recruiting a single large cohort of eligible and interested cancer survivors who meet the CRF-severity criteria and are able to commit to the intervention. However, parallel groups will also control for potentially confounding variables such as seasonal variations in Calgary, Alberta.

In summary, although there is evidence for the benefits of exercise for CRF, it is important to design interventions specifically targeting this distressing symptom, such that potential benefits are optimised. If a tailored intervention confers some benefit above a more general exercise program in cancer survivors with persistent CRF, referral to a clinical exercise physiologist should be considered as a treatment option given the suggestion that the profession can assist oncologists in the management of fatigue [130]. To our knowledge, this will be the first study to compare the effects of a traditional vs. tailored exercise intervention on CRF in fatigued cancer survivors. Using physiological, behavioural and patient-reported outcomes, this study will add to the current knowledge about both the factors contributing to CRF, and the potential reduction in CRF severity with an exercise intervention, with the ultimate objective of improving the quality of life of cancer survivors.

## Additional file

Additional file 1: SPIRIT 2013 Checklist: Recommended items to address in a clinical trial protocol. (DOC 125 kb)

## Abbreviations

ACSM: American College of Sports Medicine
AUDIT: Alcohol Use Disorders Identification Test
BF: Biceps femoris
BPI-sf: Brief Pain Inventory Short Form
CES-D: Center for Epidemiological Studies on Depression Scale
CRF: Cancer-related fatigue
CSA: Cross-sectional area
CSEP-CEP: Canadian Society for Exercise Physiology Certified Exercise Physiologist
DXA: Dual-energy X-ray absorptiometry
ECG: Electrocardiogram
EMG: Electromyography
ESAS-r: Edmonton Symptom Assessment System (revised version)
FACIT-F: Functional Assessment of Chronic Illness Therapy Fatigue Scale
FACT-G: Functional Assessment of Cancer Therapy – General
FNES: Femoral nerve electrical stimulation
GLTEQ: Godin Leisure-Time Exercise Questionnaire
HR: Heart rate
HRQL: Health-related quality of life
HRV: Heart rate variability
ISI: Insomnia severity index
MVC: Maximal voluntary contraction
PAR-Q+: Physical Activity Readiness Questionnaire for Everyone
RF: Rectus femoris
RMS: Root mean square
RPE: Rating of perceived exertion
SIT: Superimposed twitch
SP: Silent period
SPS: Social Provision Scale
TMS: Transcranial magnetic stimulation
VA: Voluntary activation
VL: Vastus lateralis
$\dot{V}{\mathrm{O}}_{2\max }$: Maximal oxygen uptake
$\dot{V}{\mathrm{O}}_{2\mathrm{peak}}$: Peak oxygen uptake
